# Multivariate association analysis of the components of metabolic syndrome from the Framingham Heart Study

**DOI:** 10.1186/1753-6561-3-s7-s42

**Published:** 2009-12-15

**Authors:** Allison R Baker, Robert J Goodloe, Emma K Larkin, Dan J Baechle, Yeunjoo E Song, Lynette S Phillips, Courtney L Gray-McGuire

**Affiliations:** 1 Department of Epidemiology and Biostatistics, Division of Genetic and Molecular Epidemiology, Case Western Reserve University, 11400 Euclid Avenue, Suite 260, Cleveland, Ohio 44106, USA; 2 Center for Clinical Investigation, Case Western Reserve University, 11400 Euclid Avenue, Suite 260, Cleveland, Ohio 44106, USA

## Abstract

Metabolic syndrome, by definition, is the manifestation of multiple, correlated metabolic impairments. It is known to have both strong environmental and genetic contributions. However, isolating genetic variants predisposing to such a complex trait has limitations. Using pedigree data, when available, may well lead to increased ability to detect variants associated with such complex traits. The ability to incorporate multiple correlated traits into a joint analysis may also allow increased detection of associated genes. Therefore, to demonstrate the utility of both univariate and multivariate family-based association analysis and to identify possible genetic variants associated with metabolic syndrome, we performed a scan of the Affymetrix 50 k Human Gene Panel data using 1) each of the traits comprising metabolic syndrome: triglycerides, high-density lipoprotein, systolic blood pressure, diastolic blood pressure, blood glucose, and body mass index, and 2) a composite trait including all of the above, jointly. Two single-nucleotide polymorphisms within the cholesterol ester transfer protein (*CETP*) gene remained significant even after correcting for multiple testing in both the univariate (*p *< 5 × 10^-7^) and multivariate (*p *< 5 × 10^-9^) association analysis. Three genes met significance for multiple traits after correction for multiple testing in the univariate analysis, while five genes remained significant in the multivariate association. We conclude that while both univariate and multivariate family-based association analysis can identify genes of interest, our multivariate approach is less affected by multiple testing correction and yields more significant results.

## Background

Although various organizations have used different criteria to define metabolic syndrome (MetSyn), it is generally agreed that MetSyn consists of a combination of impaired glucose metabolism, insulin resistance, hypertension, obesity, and dyslipidemia that increases the risk of poor cardiovascular outcomes [1]. Research, whether through heritability or association studies, suggests there is an important genetic underpinning to this disease [2]. Moreover, linkage studies have shown that analyzing the components of MetSyn as a multivariate outcome can give stronger evidence for regions harboring disease-susceptibility loci than analyses of separate univariate phenotypes [3-9].

Here we aim to establish the relationship between biomarker data for the components of MetSyn based on the World Health Organization (WHO) definition and single-nucleotide polymorphisms (SNPs) within the 50 k SNP candidate gene panel of the offspring cohort of the Framingham Heart Study (FHS), using full-pedigree information. We will compare the results of our family-based association analyses of MetSyn as a multivariate phenotype to results that consider each component of MetSyn as a univariate trait while accounting for the familial clustering of data in both analysis methods.

## Methods

### Pedigree and phenotype data

Before and during this study, all authors signed and complied with the Data Use Agreement for the Framingham Heart Study data and the Case Western Reserve University IRB. Due to large amounts of missing data for the variables of interest in the FHS original cohort, only phenotype data for individuals from the offspring cohort were included in the analyses.

However, the family structure from all cohorts was utilized in the analyses. Analyses were restricted to measurements reported from the seventh visit of the offspring cohort because more variability in the quantitative traits was expected in the older study participants. Our study was restricted to non-smokers to remove confounding by smoking status. We further trimmed our data set to reduce computational complexity by removing four pedigrees with more than 200 members each, resulting in 770 individuals and 1052 sibling pairs within 334 pedigrees.

Because the data set given did not include fasting insulin levels or waist circumference, we used the WHO 1999 definition of MetSyn to choose the variables to include in our multivariate trait, including: triglycerides (TG), high-density lipoprotein (HDL), systolic blood pressure (SBP), diastolic blood pressure (DBP), blood glucose (BG), and body mass index (BMI) as defined by weight in kilograms divided by height in meters squared [10].

Preliminary modeling identified age, sex, and the interaction of age by sex as covariates to adjust for before the association analysis. To account for the blood pressure lowering effects of medication, a constant of 10 was added to SBP and a constant of five was added to DBP for those individuals who reported using anti-hypertensive medication [11]. Due to the skewness of the variables, TG, HDL, SBP, DBP, BG, and BMI were each natural log-transformed before analysis.

### Marker data

Because MetSyn has been extensively studied and many candidate genes named, we chose to analyze the 50 k candidate gene SNP panel. Prior to association analysis, mendelian inconsistencies were identified in the data using MARKERINFO (S.A.G.E. v5.4.2) and the genotypes of all individuals in a family with an inconsistency were set to missing for each given marker.

### Association analysis

We used the following regression model as implemented in ASSOC, a program in the S.A.G.E. software suite:

where for any individual *i*, with trait *y*_1_, *c*_*ji *_is any one of *n *individual specific covariates, *η*_*i *_is a random effect comprising, in our study, the sibling and individual specific errors, *z*_*i *_is a genotype indicator for the allele *A *at a diallelic locus with alleles A and B,

and the regression coefficients *y*_*j *_and *δ *are median unbiased on the original scale of measurement. We simultaneously estimate the effect of allele *A*, covariates, and the residual variance components. The likelihood is maximized numerically over all parameters, and standard errors determined. *p*-Values for the regression coefficients and the variance components, based on the likelihood ratio and Wald test, were calculated. Any SNPs for which these two tests did not agree were removed from the results reported.

Multivariate association analysis of quantitative traits was conducted using the S.A.G.E. program RELPAL, which implements the multivariate Haseman-Elston regression technique as a two-stage association and linkage analysis [12,13]. Because we were interested only in association, we performed the first-level regression only. We constructed a multivariate model using a vector of trait values, *y*_*k*_, for the *k*^th ^family. After adjusting for individual covariates, the expected value of *y*_*k*_*y*_*k*_^T ^is the variance-covariance matrix of the traits for family *k*, defined as:

where ⊗ denotes a Kronecker product, **P **is the additive polygenic variance covariance matrix, Φ represents the matrix of kinship coefficients, **E **is the environmental variance covariance matrix, and **I **is an identity matrix incorporating random error for each individual. The two-stage approach proposed by Wang and Elston incorporates both identity-by-decent (IBD) sharing and a matrix due to the additive effect of a quantitative trait locus (QTL). However, limiting the analysis to stage 1 of RELPAL by ignoring the linkage component provides a virtually identical multivariate extension to ASSOC [13]. This association test, which considers all traits jointly, results in a score test for the variance-covariance component under constrained parameterization. The test statistic is a one-sided version of the classical score test [14]. An asymptotic *p*-value is obtained using a computational approach (see S.A.G.E. user documentation) and results in a chi-square test of fixed effects on the trait mean with degrees of freedom equal to the number of variance-covariance components in the test (in our case, because we only calculate the score test, it is one).

Because the purpose of our paper is to compare these methods, we chose to report results from both analyses for possible thresholds rather than only those for a specific threshold. For the univariate analysis, we began by assuming the SNPs within a given gene had a linkage disequilibrium (LD) measure of *r*^2 ^= 0.8 (and therefore only 20% are independent) on average and adjusted for analyzing six traits, resulting in a significance threshold of *p *< 8 × 10^-6^(0.05/[(50,000*0.2)*6]). We report results significant at this threshold as well as one order of magnitude less significant and 0.001. Similarly, for the multivariate analyses, we report results significant at *p *< 5 × 10^-6^(0.05/[(50,000*0.2)]), *p *< 5 × 10^-5 ^and 0.001. Note that because the multivariate approach considers all traits simultaneously, we use a less stringent threshold.

## Results

### Univariate association

Only one SNP, located in the *TCP11L1 *gene, which codes for a T-complex protein, met the criteria of *p *< 8 × 10^-6 ^for more than one of the univariate traits of interest. Relaxing the criteria for significance by an order of magnitude resulted in two additional significant SNPs: one in the *GRID1 *(glutamate receptor) gene, expressed in the central nervous system, and one in the *STAC *(src homology 3 and cysteine-rich domain) gene, involved in neural-specific signal transduction (Table 1). *GRID1 *showed association with both BMI and HDL and *STAC *with both SBP and DBP. If we relax our criteria further to *p *< 0.001, there were 50 effects shared across traits in 27 genes, on 16 chromosomes (results not shown).

**Table 1:** Genes significant in the univariate analysis for more than one trait^a^

			*p*-value					
			
Chromosome	SNP	Gene Name	BG	TG	HDL	SBP	DBP	BMI
3	RS6797696	*STAC*	0.96	0.34	0.95	3.41 × 10^-6^	2.71 × 10^-5^	0.32
11	RS3168277	*TCP11L1*	2.26 × 10^-10^	0.068	0.87	0.50	0.03	0.39
11	RS2273553	*TCP11L1*	0.22	0.14	1.8 × 10^-4^	0.76	0.79	0.27

^a^None of the SNPs were significant in the multivariate analysis (*p *= 0.001).

### Multivariate association

We found two SNPs significant at *p *< 5 × 10^-6 ^through multivariate analysis (Table 2). The most striking of these results is for cholesterol ester transfer protein (*CETP*) on chromosome 16 (*p *< 1 × 10^-10^), which has been associated in multiple studies with HDL. Interestingly, while the univariate analysis of HDL was also highly significant (*p *= 7 × 10^-7^), no other univariate traits met even a loose definition of association at these SNPs. This suggests that the additional significance attained in the multivariate analysis is due to a cumulative effect of multiple traits when analyzed in concert. If we relax our criteria for significance to 5 × 10^-5^, a SNP within the peripheral myelin protein gene (*PMP22*), which is expressed in the peripheral nervous system, emerges as significant (Table 2). Again, relaxing the threshold for significance to 0.001 results in over 50 additional effects, only about one-fourth of which overlap with the univariate results. Of these effects, all were more significant than the univariate analysis of the same SNP.

**Table 2:** SNPs significant in the multivariate analysis and their corresponding univariate results

			*p*-value						
			
Chromosome	SNP	Gene Name	Mutivariate	Univariate BG	Univariate TG	Univariate HDL	Univariate SBP	Univariate DBP	Univariate BMI
16	RS11508026	*CETP*	1.0 × 10^-10^	0.065	0.26	7.6 × 10^-7^	0.17	0.91	0.36
16	RS3764261	*CETP*	1.0 × 10^-10^	0.65	0.816	1.34 × 10^-6^	0.52	0.656	0.156
17	RS9901139	*PMP22*	1.97 × 10^-5^	0.29	0.06	0.276	0.069	0.075	0.0087

## Discussion

As mentioned above, the most striking result found in these analyses was at *CETP*, a gene known to play a role in maintaining cholesterol homeostasis (and likely arthrosclerosis), but found to have a marked gain in significance when incorporating all components of MetSyn into the analysis. This may be due to pleiotropy but with effects modest enough for other traits that are not detectable in a univariate analysis. It may also be due to the fact that incorporating the other components of MetSyn into the analysis yields a trait that is more reflective of the biological phenomenon affected by this gene than the simple clinical measure HDL. Other results also suggest that while univariate analysis may indeed be effective in isolating genetic effects for complex traits like MetSyn, incorporating other components of such a syndrome can allow a prespecified threshold of significance to be met even if accounting only for the performance of fewer tests. Other examples of possible pleiotropy are shown in our analysis, but are not as compelling, both because the gain in significance is not as striking and the genes themselves are not such striking candidates. One such example is *LOC65358*, a locus of unknown function with a multivariate *p*-value of 0.0004 and univariate *p*-values for BG and DBP of 0.002 and 0.01, respectively. We do note, however, one instance in which the univariate result was more significant than the multivariate: *C9orf93*. This is an open reading frame on chromosome 9 and the univariate analysis of SBP and DBP in this region yielded *p*-values of 0.0001 and 0.00001, respectively, but the multivariate analysis yielded a *p*-value of 0.009. Certainly, after considering the increased number of univariate tests done, the difference in the level of significance is not marked. However, this does illustrate one case in which a gene may not be pleiotropic across the components of MetSyn or offer a more biologically relevant trait.

## Conclusion

The purpose of this study was to illustrate, in the context of family-based association analysis, the benefit of simultaneously considering the highly correlated traits that comprise MetSyn. We demonstrate that for multiple genes, one of which is known to be associated with cholesterol homeostasis, significance can be greatly increased when the other components of MetSyn are simultaneously considered. In this we find the benefits of multivariate analysis, because it can serve as a mechanism by which to control for multiple comparisons, better define a trait of interest, and aid in the detection of pleiotropic effects.

## List of abbreviations used

BG: Blood glucose; BMI: Body mass index; CETP: Cholesterol ester transfer protein; DBP: Diastolic blood pressure; FHS: Framingham Heart Study; HDL: High-density lipoprotein; IBD: Identity by decent; LD: Linkage disequilibrium; MetSyn: Metabolic syndrome; QTL: Quantitative trait locus; SBP: Systolic blood pressure; SNPs: Single-nucleotide polymorphisms; TG: Triglycerides; WHO: World Health Organization.

## Competing interests

The authors declare that they have no competing interests.

## Authors' contributions

ARB and RJG performed the multivariate analyses and drafted the manuscript. EKL helped draft the manuscript. DJB and YES prepared the data for S.A.G.E. analysis and developed the programming scheme. LSP performed the univariate analyses. CLG-M conceived of and designed the study and helped draft the manuscript. All authors read and approved the final manuscript.
